# Frequent Epigenetic Silencing of the Folate-Metabolising Gene *Cystathionine-Beta-Synthase* in Gastrointestinal Cancer

**DOI:** 10.1371/journal.pone.0049683

**Published:** 2012-11-13

**Authors:** Hong Zhao, Qinshan Li, Jian Wang, Xianwei Su, Ka Man Ng, Tian Qiu, Ling Shan, Yun Ling, Linfang Wang, Jianqiang Cai, Jianming Ying

**Affiliations:** 1 Department of Abdominal Surgical Oncology, Cancer Hospital, Peking Union Medical College & Chinese Academy of Medical Sciences, Beijing, China; 2 National Laboratory of Medical Molecular Biology, Institute of Basic Medical Sciences, Peking Union Medical College & Chinese Academy of Medical Sciences, Beijing, China; 3 Cancer Epigenetics Laboratory, Department of Clinical Oncology, State Key Laboratory of Oncology in South China, Sir YK Pao Center for Cancer and Li Ka Shing Institute of Health Sciences, The Chinese University of Hong Kong, Hong Kong; 4 Department of Pathology, Cancer Hospital, Peking Union Medical College & Chinese Academy of Medical Sciences, Beijing, China; The Chinese University of Hong Kong, Hong Kong

## Abstract

**Background:**

Both gastric and colorectal cancers (CRC) are the most frequently occurring malignancies worldwide with the overall survival of these patients remains unsatisfied. Identification of tumor suppressor genes (TSG) silenced by promoter CpG methylation uncovers mechanisms of tumorigenesis and identifies new epigenetic biomarkers for early cancer detection and prognosis assessment. Cystathionine-beta-synthase (CBS) functions in the folate metabolism pathway, which is intricately linked to methylation of genomic DNA. Dysregulation of DNA methylation contributes substantially to cancer development.

**Methodology/Principal Findings:**

To identify potential TSGs silenced by aberrant promoter methylation in CRC, we analyzed tumor and adjacent tissues from CRC cases using the Illumina Human Methylation45 BeadChip. We identified hypermethylation of the *CBS* gene in CRC samples, compared to adjacent tissues. Methylation and decreased mRNA expression of *CBS* were detected in most CRC cell lines by methylation-specific PCR and semiquantitative RT-PCR, as well as in gastric cancer. Treatment with 5-aza-2'-deoxycytidine and/or trichostatin A reversed methylation and restored *CBS* mRNA expression indicating a direct effect. Aberrant methylation was further detected in 31% of primary CRCs (29 of 96) and 55% of gastric tumors (11 of 20). In contrast, methylation was seldom found in normal tissues adjacent to the tumor. *CBS* methylation was associated with *KRAS* mutations in primary CRCs (*P* = 0.04, by χ^2^-test). However, no association was found between *CBS* methylation or *KRAS* mutations with cancer relapse/metastasis in Stage II CRC patients.

**Conclusion:**

A novel finding from this study is that the folate metabolism enzyme *CBS* mRNA levels are frequently downregulated through CpG methylation of the *CBS* gene in gastric cancer and CRC, suggesting that *CBS* functions as a tumor suppressor gene. These findings warrant further study of *CBS* as an epigenetic biomarker for molecular diagnosis of gastrointestinal cancers.

## Introduction

Both gastric and colorectal cancers are the most frequently occurring malignancies worldwide whose incidence has increased in recent years [1], [2]. Although diverse novel treatment modalities, including surgical, medical, and radiological, have been introduced, the clinical course of gastric and colorectal cancer (CRC) is variable and the overall prognosis remains unsatisfactory. Therefore, identification of key genes involved in the molecular pathogenesis of gastric cancer and CRC are likely to result in novel and more effective therapeutic strategies.

Inactivation of multiple tumor suppressor genes (TSG) is a key molecular event in the multi-step genetic pathogenesis of CRC [3]. In addition to genetic changes, epigenetic inactivation of TSGs plays an important role in carcinogenesis [4]. Epigenetic silencing through aberrant methylation of CpG islands (CGI) in TSG promoter regions occurs in virtually all tumor types [4]. Particularly, a growing list of aberrantly methylated TSGs has been reported in CRCs, including *APC*, *MGMT*, *MLH1*, *p16^INK4A^*, *VHL*, *RASSF1A*, *HIC1*, *CHFR*, *ADAMTS18*, *PCDH10* and *DLEC1* [5]–[10] as well as in gastric cancer [11], [12].

In the present study, we screened for TSGs silenced by aberrant promoter methylation in CRC and found hypermethylation of the *cystathionine-beta-synthase* (*CBS*) gene which encodes for a key enzyme in folate metabolism [13], [14]. Recent work has focused on enzymes involved in folate metabolism, since methylation of DNA is dependent on these pathways [15]–[19]. Genetic instability with characteristic chromosomal imbalances is a characteristic feature of carcinogenesis. Homocysteine and folate metabolism is related to DNA integrity, and genetic variants that functionally influence homocysteine and folate metabolism are associated with different types of cancer such as CRC, non-Hodgkin’s lymphoma, etc [20], [21]. The aim of the present work was to examine whether *CBS* was a new potential TSG and to test if *CBS* mRNA expression was downregulated by promoter hypermethylation in CRC, as well as in gastric cancer. We also investigated the potential role of *CBS* gene hypermethylation as a biomarker to predict tumor relapse or metastasis in stage II (T_3_N_0_M_0_) CRC patients.

## Materials and Methods

### Ethics Statement

This study was conducted in accordance with the Helsinki guidelines for human subjects studies and was approved by the Institutional Review Board of the Cancer Hospital, Chinese Academy of Medical Sciences (CAMS), Beijing, China. Signed informed consent was obtained from all study participants for sample collection and analysis.

### Cell Lines

Four CRC (HCT116, HT29, LoVo and SW480) and 16 gastric cancer (Kato III, YCC1, YCC2, YCC3, YCC6, YCC7, YCC9, YCC10, YCC11, YCC16, SNU719, AGS, MKN28, MKN45, SNU1 and SNU16) cell lines were used [10]. Cell lines were routinely maintained in RPMI-1640 medium with 10% FBS. The HCT116 cell line with genetic knockout of DNA methyl-transferase genes *DNMT1* and *DNMT3B* (double knockout, HCT116^DKO^) was a gift from Dr. Bert Vogelstein, Johns Hopkins University [22] and cultured in the presence of 0.4 mg/ml genecitin or 0.05 mg/ml hygromycin.

### Tumor Samples

All primary samples including four paired fresh CRC and normal tissues, 96 primary formalin fixed paraffin embedded (FFPE) CRC tissues and 20 FFPE gastric cancer of tissues, were obtained from the Department of Pathology, Cancer Hospital, CAMS, Beijing, China. In addition, for methylation study, corresponding tumor-free margins from 20 gastric cancer and 17 CRC patients were analyzed. All patients did not undergo previous chemo- or radiotherapy since they presented with resectable tumors. Fresh specimens were snap-frozen in liquid N_2_ and subsequently stored at −80°C until processed. The diagnosis was confirmed through hematoxylin and eosin (HE)-staining and histopathological analysis was performed to define representative tumor regions. Clinical information was available for all CRC patients, including gender, age, differentiation, and follow-up data. The median age of CRC patients was 68 years (range 37–93), and male to female ratio was 1.4∶1 (56∶40). The number of well, moderate and poor-differentiation cases was 14, 56 and 26, respectively. Cancer staging (pTNM) was defined according to the 7^th^ edition of American Joint Committee of Cancer [23]. All CRC patients are at stage II (pT_3_N_0_M_0_).

### DNA and RNA Extraction

Total RNA and DNA were extracted from cell lines using the TRI Reagent (Molecular Research Centre). For fresh tumor tissues, frozen sections were obtained and reviewed to make sure that tumor content was more than 80% of section area. DNA was extracted from fresh tissues and FFPE samples using the QIAamp® DNA Mini kit (Qiagen) following the manufacturer’s instructions.

### Pharmacologic Demethylation

Two gastric cancer cell lines (YCC10 and SNU719) and one CRC (HCT116) cell line with silenced *CBS* were treated with 5 uM of 5-aza-2′-deoxycytidine (Aza) (Sigma) for three days and followed with 100 nM trichostatin A (TSA, Cayman) for one day as described previously [24]. After treatment, total DNA and RNA was extracted.

### DNA Methylation Analysis

All DNA specimens were subjected to bisulfite modification using the EZ DNA Methylation Kit (Zymo Research) according to the manufacturer instructions. One µg of genomic DNA from each sample was bisulfite converted and eluted in 18 µl elution buffer, and 5 µl of each sample was analyzed with the Illumina Infinium DNA methylation assay using the Human Methylation45 BeadChip (Infinium Methylation 450 K, Illumina), which is an allele-specific assay with 480,000 CpG loci covering the whole genome [25]. The protocols and probe information are available at www.illumina.com. The results of the DNA methylation assay were compiled for each locus using the Illumina Bead Studio software (Illumina) and are reported as beta (β) values which are DNA methylation scores ranging from 0 to 1,reflecting the fractional DNA methylation level of a single CpG site [25].

### Semi-quantitative Reverse Transcription PCR (RT-PCR)

RT–PCR was performed as described previously [9], using *GAPDH* as control. The primers for *CBS* are listed in Table 1. The PCR program utilized an initial denaturation at 95°C for 10 min, 35 cycles (94°C for 30 s, 55°C for 30 s and 72°C for 30 s), and a final extension step at 72°C for 10 min.

**Table 1:** Primers used in this study.

PCR	Primer	Sequence	Productsize (bp)	PCRcycles	Annealingtemp. (°C)
RT-PCR	CBS-F	CAGAGGATAAGGAAGCCAAG	192	35	55
	CBS-R	TCCCAATCTTGTTGATTCTGAC			
MSP	CBSm3	CGAGATATTGGTCGGCGTC	129	40	60
	CBSm4	CCCCGACTACGACGAAACG			
	CBSu3	TTATGAGATATTGGTTGGTGTT	135	41	58
	CBSu4	TACCCCAACTACAACAAAACA			

### *KRAS* Mutation Detection by Real-time PCR

The seven hotspot mutations within codon 12 and 13 of *KRAS* gene were examined using the Human *KRAS* Mutation Qualitative Detection Kit (ACCB Biotech Ltd.). Fluorescent probes were designed to specifically detect 7 different point mutations (G12V/S/R/D/A/C and G13D). The assay was carried out according to the manufacturer’s protocol using the MX3000P real-time PCR system (Stratagene). Presence or absence of mutations was assessed qualitatively from the fluorescence amplification curve.

### Bisulphite Treatment and Methylation-specific PCR (MSP) Analysis

Bisulphite modification of DNA was carried out as described earlier using 2.4M sodium metabisulphite [26]. MSP was conducted as previously described [9]. MSP primers are listed in Table 1. MSP PCR primer specificity was confirmed as they did not amplify non-bisulphite-treated genomic DNA templates. The MSP products of selected samples were confirmed by direct sequencing.

### Statistical Analysis

χ^2^ tests were used to analyze possible correlations between clinical parameters, *KRAS* mutation and *CBS* methylation status of tumor samples. All analyses were performed using SPSS for Windows. *P*<0.05 was considered statistically significant.

## Results

### Identification of *CBS* as a Hypermethylated Gene in CRC

**Figure 1:**
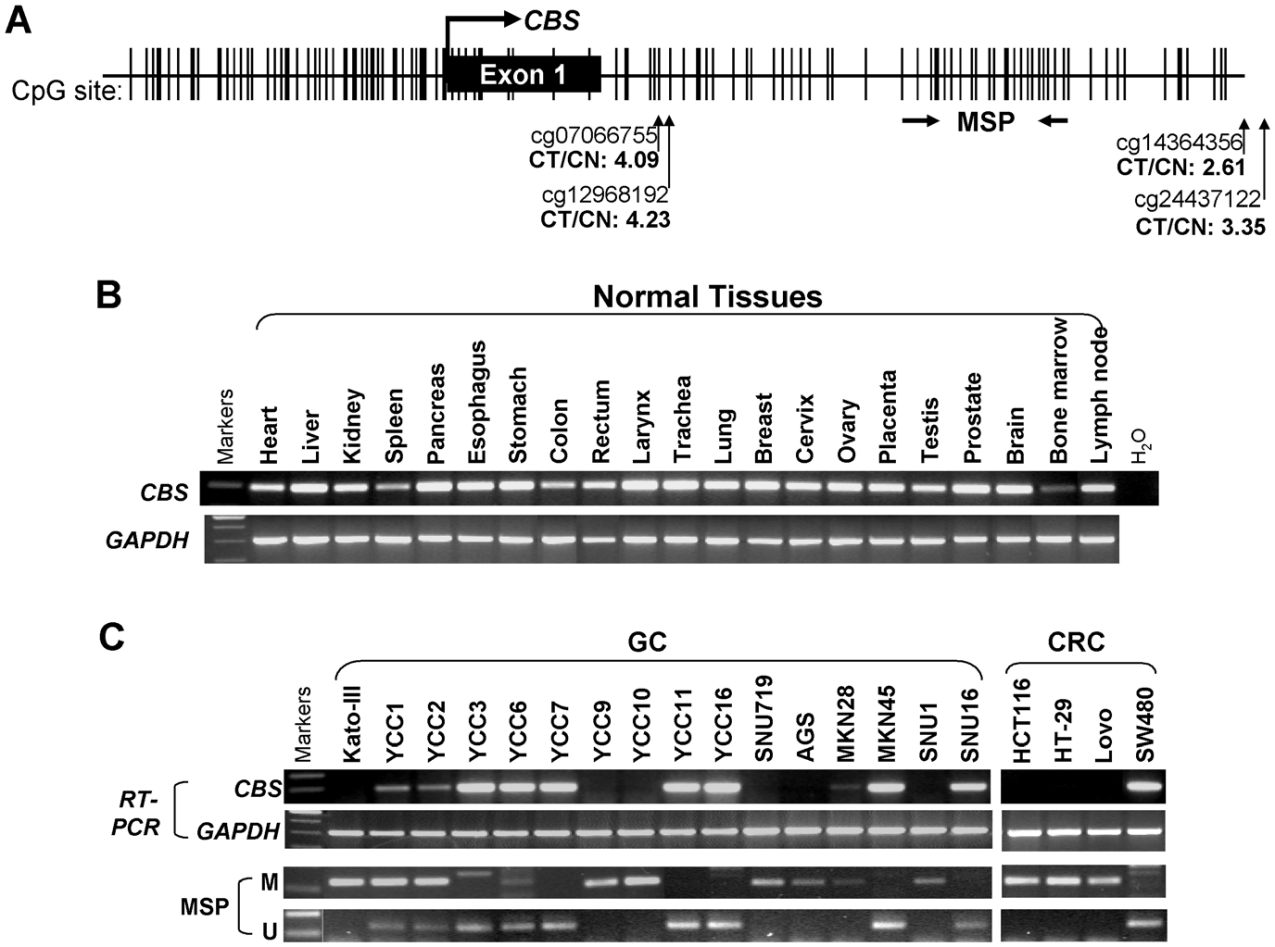
Frequent downregulation of *CBS* by promoter methylation in gastric and colorectal cancer cell lines. *A*, schematic diagram of the *CBS* promoter CpG island (CGI), with exon 1 (black rectangle), CpG sites (short vertical lines), MSP region is shown. The transcription start site is indicated by a curved arrow. The four CpG sites located in the *CBS* gene region identified by Illumina methylation array to be significantly hypermethylated in tumor compared to non-tumor tissues (CT/CN) are shown. *B*, Expression of *CBS* was readily detected in normal tissues. *C*, Expression and methylation of *CBS* in cancer cell lines were examined by RT-PCR and MSP, with *GAPDH* as a control. M, methylated, U, unmethylated.

We analyzed tumor and adjacent tissues from four Chinese CRC cases using Illumina methylation array that screen for 480,000 CpG *loci* covering the whole genome. Among all CpG sites that were hypermethylated in tumor samples (data not shown), four CpG sites located were in the *CBS* gene (Figure 1A). Analysis of the genomic sequence of the *CBS* gene, revealed that its putative promoter, exon 1 and intron 1 was a typical CGI and thus susceptible to epigenetic silencing (Figure 1A) [27].

### Frequent Silencing of *CBS* by Promoter Methylation in Multiple CRC and Gastric Cancer Cell Lines

Previously, *CBS* has been shown to be strongly expressed in the brain, as well as liver, pancreas, kidney and fetal tissues [13]. To further examine the correlation between *CBS* methylation and mRNA expression, we first investigated a panel of normal human tissues by semiquantitative RT-PCR. Our results showed that *CBS* was expressed in all normal tissues including colon, rectum and stomach, though at different expression levels. The highest expression level was found in pancreas and respiratory system tissues (larynx, trachea and lung) whereas low expression was in bone marrow tissue (Figure 1B).

**Figure 2:**
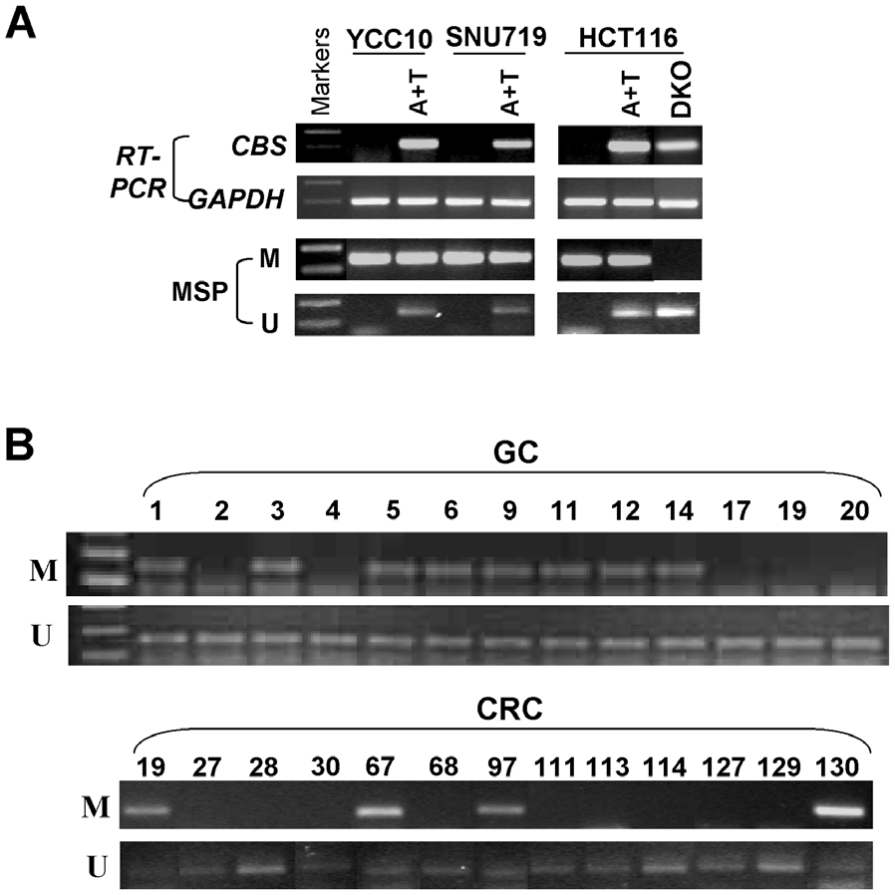
Epigenetic downregulation of *CBS* in gastrointestinal tract cancer. *A*, Pharmacologic or genetic demethylation using Aza with TSA induces *CBS* expression in methylated and silenced cell lines. A+T: Aza and TSA treatment. *B*, Representative MSP results of *CBS* methylation in primary gastric cancer (GC) and colorectal cancer (CRC). U: MSP for unmethylated promoter, M: MSP for methylated promoter.

To contrast *CBS* expression in normal tissues to malignant cells, we analyzed gastrointestinal tumor cell lines. Semiquantitative RT-PCR showed that *CBS* expression was decreased or silenced in 56.3% (9/16) of gastric cancer and 75.0% (3/4) of CRC cell lines (Figure 1C). *CBS* methylation status was also analyzed by MSP in these cell lines. We found that cell lines with reduced or silenced *CBS* expression had methylated promoters, whereas no methylation was found in the normal *CBS* expression cell lines (Figure 2B) indicating that *CBS* promoter methylation is a major mechanism for transcriptional silencing of this gene in most of the examined CRC and gastric cancer cell lines.

### Restoration of *CBS* Expression by Pharmacologic and Genetic Demethylation

To determine whether methylation directly mediates the decrease of *CBS* mRNA expression, one methylated CRC (HCT116) and two methylated gastric cancer (YCC10 and SNU719) cell lines were treated with Aza, a DNA methyltransferase inhibitor, and TSA. After treatment, *CBS* expression was restored in methylated cell lines along with a marked increase of unmethylated promoter alleles (Figure 2A). We also found that *CBS* could be reactivated in the HCT116^DKO^ CRC cell line which is genetically demethylated through double knockout of both DNMT1 and DNMT3B. Concomitantly, unmethylated *CBS* alleles were detected in Aza-treated and HCT116^DKO^ cells, indicating that methylation of the *CBS* promoter directly leads to its silencing in CRC and gastric cancers.

### Frequent *CBS* Methylation in Primary CRC and Gastric Tumors

We further investigated the presence of *CBS* promoter methylation in 96 primary CRC and 20 gastric cancer samples using MSP analysis. *CBS* promoter methylation was detected in 30% of CRC (29/96) and 55% of gastric tumors (11 of 20), but was infrequently found in normal gastric (1/20, 5%) or colon tissue (0/17, 0%) samples (Figure 2B). There was no significant association of *CBS* methylation with gender, age or tumor differentiation in CRC patients (data not shown). These results suggest that hypermethylation of the *CBS* promoter is a common event in CRC and gastric cancers.

### Correlation of *CBS* Methylation and *KRAS* Mutation in Primary CRC Tumors

Common mutations of codons 12 and 13 at the exon 2 of the *KRAS* gene were examined in 96 primary CRC, where *CBS* methylation status was previously analyzed (**Table 2**). *KRAS* mutation was detected in 45% (13/29) of *CBS*-methylated and 24% (16/67) of *CBS*-unmethylated primary CRCs indicating a positive correlation between *CBS* methylation and *KRAS* mutations (*P*<0.05). Further analysis showed no correlation of *CBS* methylation and *KRAS* mutation with cancer relapse/metastasis in stage II CRC patients (**Table 2**).

**Table 2:** Correlation of *CBS* methylation, *KRAS* mutation and tumor relapse/metastasis status in primary stage II CRC samples.

		CBSmethylation				CBSmethylation				Replase/metastasis	
		Yes	No			Yes	No			Yes	No
KRAS mutation	Yes	13	16	Replase/metastasis	Yes	14	26	KRAS mutation	Yes	15	14
	No	16	51		No	15	41		No	25	42
P value		0.04		P value		0.388		P value		0.189	

## Discussion

To our knowledge, this is the first report to identify *CBS* as a methylated candidate TSG in gastrointestinal cancers. We demonstrated that *CBS* mRNA expression was absent in several colorectal and gastric cancer cell lines due to promoter methylation. Furthermore, the *CBS* gene was frequently methylated in CRC and gastric cancer patients, thus suggesting that *CBS* acts as a TSG in CRC and gastric cancer being frequently inactivated by methylation.

The gene *CBS*, located on chromosome 21q22.3, encodes an important enzyme involved in transulfuration of homocysteine produced during methyl-group metabolism [27]. Notably, *CBS* deficiency causes increased plasma methionine levels and decreased cysteine levels [14], [28] which in turn are known to correlate with homocystinuria, cardiovascular disease and hepatocellular carcinoma [14], [21]. The transulfuration pathway links methionine metabolism to the biosynthesis of cellular redox-controlling molecules such as cysteine, glutathione, and taurine [18]. Cysteine generated through the transulfuration pathway determines cellular redox-controlling molecule levels, such as glutathione and taurine protecting cells against reactive species-induced damage [29] of DNA through base and sugar modifications, base-free sites, DNA-protein crosslinks, and strand breaks [30], [31]. Thus, downregulated expression of *CBS* may impair the production of glutathione thus facilitating tumorigenesis [16]. In addition, redox imbalance stimulates protein kinase and poly-(ADP ribosylation) pathways leading to inhibition of apoptosis and resulting in necrotic cell death, followed by inflammatory responses and tumor development [32]. There are some studies that have evaluated the association between malignant tumor susceptibility and polymorphisms of the *CBS* gene (844ins68) in CRC [19], [33] but not in esophageal or gastric cancer [15]. In addition, *CBS* 844ins68 polymorphism is associated with decreased survival in head and neck squamous cell cancer [34].

Furthermore, the loss of *CBS* expression could lead to the accumulation of homocysteine, which will be recycled to methionine by methionine systhase via the remethylation pathway [14]. As methionine acts as the source of methyl group donor for DNA methylation, its increase caused by loss of *CBS* expression may dysregulate DNA methylation. Our results revealed frequent methylation of *CBS* in gastrointestinal cancer cell lines and primary tumors. Thus, the suppression of *CBS* by promoter methylation, instead of genetic alterations, would also result in a disturbance of methyl-group metabolism and contribute to cancer development with increased DNA damage by oxidative stress, impairing antioxidant capacity, and dysregulating DNA methylation. However, *CBS* methylation was not associated with recurrence in our cohort of patients with stage II CRC. We suggest that larger cohorts of patients are necessary to study this potential association with sufficient statistical power.

The prognostic value of *KRAS* mutations in patients with CRC remains controversial. A study by Roth *et al.* suggested that the prognostic value for *KRAS* mutation status for PFS and OS was lacking in patients with stage II and III resected colon cancer [35]. However, it has been reported that stage III patients having *KRAS* mutations displayed significantly worse disease-free survival compared with those having wild-type *KRAS* [36]. More importantly, few studies have differentiated *KRAS* mutations at codon 12 from those at codon 13 with respect to clinicopathological features and survival [37]–[40]. In this study, we could not identify *KRAS* mutation status as a prognostic factor for relapse or metastasis in patients with stage II resected CRC. Instead, we found that *CBS* methylation was significantly associated with *KRAS* mutations. This feature is in line with a previous report showing that CpG island methylator phenotype (CIMP) is associated with *KRAS* mutations [41].

In summary, the salient finding from this study is that *CBS* is suppressed by promoter methylation in colorectal and gastric cancers. We found that the methylation-mediated silencing of *CBS* could be reversed by genetic or pharmacologic demethylation, suggesting that *CBS* functions as a tumor suppressor in these cancer types. The deregulation of *CBS* and its association with a malignant tumor phenotype is potentially associated to the crucial role of CBS in methionine metabolism and the maintenance of intracellular redox homeostasis. Our study warrants further analysis of *CBS* as an epigenetic biomarker for the molecular diagnosis of CRC and gastric cancer.

## References

[1] Crew KD, Neugut AI (2006) Epidemiology of gastric cancer. World J Gastroenterol 12: 354–362. 16489633 10.3748/wjg.v12.i3.354 PMC4066052

[2] Jemal A, Bray F (2011) Center MM, Ferlay J, Ward E, et al (2011) Global cancer statistics. CA Cancer J Clin 61: 69–90. 21296855 10.3322/caac.20107

[3] Kinzler KW, Vogelstein B (1996) Lessons from hereditary colorectal cancer. Cell 87: 159–170. 8861899 10.1016/s0092-8674(00)81333-1

[4] Jones PA, Baylin SB (2002) The fundamental role of epigenetic events in cancer. Nat Rev Genet 3: 415–428. 12042769 10.1038/nrg816

[5] Esteller M, Risques RA, Toyota M, Capella G, Moreno V, et al (2001) Promoter hypermethylation of the DNA repair gene O(6)-methylguanine-DNA methyltransferase is associated with the presence of G:C to A:T transition mutations in p53 in human colorectal tumorigenesis. Cancer Res 61: 4689–4692. 11406538

[6] Herman JG, Umar A, Polyak K, Graff JR, Ahuja N, et al (1998) Incidence and functional consequences of hMLH1 promoter hypermethylation in colorectal carcinoma. Proc Natl Acad Sci U S A 95: 6870–6875. 9618505 10.1073/pnas.95.12.6870 PMC22665

[7] Jin H, Wang X, Ying J, Wong AH, Cui Y, et al (2007) *Epigenetic silencing of a Ca(2+)-regulated Ras* GTPase-activating protein RASAL defines a new mechanism of Ras activation in human cancers. Proc Natl Acad Sci U S A 104: 12353–12358. 17640920 10.1073/pnas.0700153104 PMC1941473

[8] Kim BN, Yamamoto H, Ikeda K, Damdinsuren B, Sugita Y, et al (2005) Methylation and expression of p16INK4 tumor suppressor gene in primary colorectal cancer tissues. Int J Oncol 26: 1217–1226. 15809712

[9] Ying J, Li H, Seng TJ, Langford C, Srivastava G, et al (2006) Functional epigenetics identifies a protocadherin PCDH10 as a candidate tumor suppressor for nasopharyngeal, esophageal and multiple other carcinomas with frequent methylation. Oncogene 25: 1070–1080. 16247458 10.1038/sj.onc.1209154

[10] Ying J, Poon FF, Yu J, Geng H, Wong AH, et al (2009) DLEC1 is a functional 3p22.3 tumour suppressor silenced by promoter CpG methylation in colon and gastric cancers. Br J Cancer 100: 663–669. 19156137 10.1038/sj.bjc.6604888 PMC2653732

[11] Leung WK, Yu J, Ng EK, To KF, Ma PK, et al (2001) Concurrent hypermethylation of multiple tumor-related genes in gastric carcinoma and adjacent normal tissues. Cancer 91: 2294–2301. 11413518

[12] Bernal C, Aguayo F, Villarroel C, Vargas M, Díaz I, et al (2008) Reprimo as a potential biomarker for early detection in gastric cancer. Clin Cancer Res 14: 6264–6269. 18829507 10.1158/1078-0432.CCR-07-4522

[13] Bao L, Vlcek C, Paces V, Kraus JP (1998) Identification and tissue distribution of human cystathionine beta-synthase mRNA isoforms. Arch Biochem Biophys 350: 95–103. 9466825 10.1006/abbi.1997.0486

[14] Kraus JP, Janosík M, Kozich V, Mandell R, Shih V, et al (1999) Cystathionine beta-synthase mutations in homocystinuria. Hum Mutat 13: 362–375. 10338090 10.1002/(SICI)1098-1004(1999)13:5<362::AID-HUMU4>3.0.CO;2-K

[15] Ott N, Geddert H, Sarbia M (2008) Polymorphisms in methionine synthase (A2756G) and cystathionine beta-synthase (844ins68) and susceptibility to carcinomas of the upper gastrointestinal tract. J Cancer Res Clin Oncol 134: 405–410. 17726616 10.1007/s00432-007-0301-2 PMC12161651

[16] Parl FF (2005) Glutathione S-transferase genotypes and cancer risk. Cancer Lett 221: 123–129. 15808397 10.1016/j.canlet.2004.06.016

[17] Paz MF, Avila S, Fraga MF, Pollan M, Capella G, et al (2002) Germ-line variants in methyl-group metabolism genes and susceptibility to DNA methylation in normal tissues and human primary tumors. Cancer Res 62: 4519–4524. 12154064

[18] Rosado JO, Salvador M, Bonatto D (2007) Importance of the trans-sulfuration pathway in cancer prevention and promotion. Mol Cell Biochem 301: 1–12. 17180248 10.1007/s11010-006-9389-y

[19] Shannon B, Gnanasampanthan S, Beilby J, Iacopetta B (2002) A polymorphism in the methylenetetrahydrofolate reductase gene predisposes to colorectal cancers with microsatellite instability. Gut 50: 520–524. 11889073 10.1136/gut.50.4.520 PMC1773174

[20] Linnebank M, Schmidt S, Kölsch H, Linnebank A, Heun R, et al (2004) The methionine synthase polymorphism D919G alters susceptibility to primary central nervous system lymphoma. Br J Cancer 90: 1969–1971. 15138479 10.1038/sj.bjc.6601777 PMC2409477

[21] Kim J, Hong SJ, Park JH, Park SY, Kim SW, et al (2009) Expression of cystathionine beta-synthase is downregulated in hepatocellular carcinoma and associated with poor prognosis. Oncol Rep 21: 1449–1454. 19424622 10.3892/or_00000373

[22] Rhee I, Bachman KE, Park BH, Jair KW, Yen RW, et al (2002) DNMT1 and DNMT3b cooperate to silence genes in human cancer cells. Nature 416: 552–556. 11932749 10.1038/416552a

[23] Edge SE, B.D., Carducci MA, Compton CA (2010) AJCC Cancer Staging Manual. 7th Ed., New York: Springer.

[24] Ying J, Srivastava G, Hsieh WS, Gao Z, Murray P, et al (2005) The stress-responsive gene GADD45G is a functional tumor suppressor, with its response to environmental stresses frequently disrupted epigenetically in multiple tumors. Clin Cancer Res 11: 6442–6449. 16166418 10.1158/1078-0432.CCR-05-0267

[25] Dedeurwaerder S, Defrance M, Calonne E, Denis H, Sotiriou C, et al (2011) Evaluation of the Infinium Methylation 450K technology. Epigenomics 3: 771–784. 22126295 10.2217/epi.11.105

[26] Tao Q, Huang H, Geiman TM, Lim CY, Fu L, et al (2002) Defective de novo methylation of viral and cellular DNA sequences in ICF syndrome cells. Hum Mol Genet 11: 2091–2102. 12189161 10.1093/hmg/11.18.2091

[27] Dutta S, Sinha S, Chattopadhyay A, Gangopadhyay PK, Mukhopadhyay J, et al (2005) Cystathionine beta-synthase T833C/844INS68 polymorphism: a family-based study on mentally retarded children. Behav Brain Funct 1: 25. 16375773 10.1186/1744-9081-1-25 PMC1334203

[28] Majors AK, Pyeritz RE (2000) Pyeritz, A deficiency of cysteine impairs fibrillin-1 deposition: implications for the pathogenesis of cystathionine beta-synthase deficiency. Mol Genet Metab 70: 252–260. 10993712 10.1006/mgme.2000.3024

[29] Prudova A, Bauman Z, Braun A, Vitvitsky V, Lu SC, et al (2006) S-adenosylmethionine stabilizes cystathionine beta-synthase and modulates redox capacity. Proc Natl Acad Sci U S A 103: 6489–6494. 16614071 10.1073/pnas.0509531103 PMC1458911

[30] Demple B, Harrison L (1994) Repair of oxidative damage to DNA: enzymology and biology. Annu Rev Biochem 63: 915–948. 7979257 10.1146/annurev.bi.63.070194.004411

[31] Laval J (1996) Role of DNA repair enzymes in the cellular resistance to oxidative stress. Pathol Biol (Paris) 44: 14–24. 8734295

[32] Audebert M, Salles B, Calsou P (2004) Involvement of poly(ADP-ribose) polymerase-1 and XRCC1/DNA ligase III in an alternative route for DNA double-strand breaks rejoining. J Biol Chem 279: 55117–55126. 15498778 10.1074/jbc.M404524200

[33] Le Marchand L, Donlon T, Hankin JH, Kolonel LN, Wilkens LR, et al (2002) B-vitamin intake, metabolic genes, and colorectal cancer risk (United States). Cancer Causes Control 13: 239–248. 12020105 10.1023/a:1015057614870

[34] Galbiatti AL, da Silva LM, Ruiz-Cintra MT, Raposo LS, Maníglia JV, et al (2012) Association between 11 genetic polymorphisms in folate-metabolising genes and head and neck cancer risk. Eur J Cancer 48: 1525–1531. 22051736 10.1016/j.ejca.2011.09.025

[35] Roth AD, Tejpar S, Delorenzi M, Yan P, Fiocca R, et al (2010) Prognostic role of KRAS and BRAF in stage II and III resected colon cancer: results of the translational study on the PETACC-3, EORTC 40993, SAKK 60–00 trial. J Clin Oncol 28: 466–474. 20008640 10.1200/JCO.2009.23.3452

[36] Fariña-Sarasqueta A, van Lijnschoten G, Moerland E, Creemers GJ, Lemmens VE, et al (2010) The BRAF V600E mutation is an independent prognostic factor for survival in stage II and stage III colon cancer patients. Ann Oncol 21: 2396–2402. 20501503 10.1093/annonc/mdq258

[37] Ahn JB, Chung WB, Maeda O, Shin SJ, Kim HS, et al (2011) DNA methylation predicts recurrence from resected stage III proximal colon cancer. Cancer 117: 1847–1854. 21509761 10.1002/cncr.25737 PMC3117123

[38] Andreyev HJ, Norman AR, Cunningham D, Oates J, Dix BR, et al (2001) Kirsten ras mutations in patients with colorectal cancer: the ‘RASCAL II’ study. Br J Cancer 85: 692–696. 11531254 10.1054/bjoc.2001.1964 PMC2364126

[39] Bennecke M, Kriegl L, Bajbouj M, Retzlaff K, Robine S, et al (2010) Ink4a/Arf and oncogene-induced senescence prevent tumor progression during alternative colorectal tumorigenesis. Cancer Cell 18: 135–146. 20708155 10.1016/j.ccr.2010.06.013

[40] Guerrero S, Casanova I, Farré L, Mazo A, Capellà G, et al (2000) K-ras codon 12 mutation induces higher level of resistance to apoptosis and predisposition to anchorage-independent growth than codon 13 mutation or proto-oncogene overexpression. Cancer Res 60: 6750–6756. 11118062

[41] Zlobec I, Bihl M, Foerster A, Rufle A, Lugli A (2011) Comprehensive analysis of CpG island methylator phenotype (CIMP)-high, -low, and -negative colorectal cancers based on protein marker expression and molecular features. J Pathol 225: 336–343. 21660972 10.1002/path.2879

